# Transfer Accuracy in Digital Indirect Bonding: A Methodological Umbrella Review of Definitions, Measurement Frameworks, and Evidence Synthesis

**DOI:** 10.3390/bioengineering13060607

**Published:** 2026-05-23

**Authors:** Elisabetta Lalli, Alessio Verdecchia, Simone Parrini, Gabriele Rossini, Federico Ezequiel Malagraba, María Mónica Beti, Edoardo Marchese, Enrico Spinas

**Affiliations:** 1 Department of Surgical Sciences, Postgraduate School in Orthodontics, University of Cagliari, 09124 Cagliari, Italy; dottoressa.lalli@gmail.com (E.L.); edoardo.marchese@inwind.it (E.M.); enricospinas@tiscali.it (E.S.); 2 Orthodontics Division, Instituto Asturiano de Odontología, Universidad de Oviedo, 33006 Oviedo, Spain; 3 Department of Orthodontics, University of Siena, 53100 Siena, Italy; 4 Private Practice, 20100 Milan, Italy; dr.gabriele.rossini@gmail.com; 5 Orthodontics Division, Universidad Católica de La Plata, La Plata 1900, Argentina; federicomalagraba@gmail.com (F.E.M.); odmbeti@gmail.com (M.M.B.)

**Keywords:** indirect bonding, transfer accuracy, orthodontics, methodological umbrella review, outcome measurement, bracket placement accuracy, measurement heterogeneity, reference systems, digital orthodontics, evidence synthesis

## Abstract

Transfer accuracy is widely used to evaluate orthodontic indirect bonding workflows, particularly in the context of digital CAD/CAM planning and three-dimensional bracket positioning. However, substantial heterogeneity in its definition, measurement, and reporting may limit comparability and clinical interpretability across systematic reviews. This methodological umbrella review examined how transfer accuracy is operationalized as an outcome construct, with specific focus on conceptual definitions, dimensional frameworks, reference systems, measurement pipelines, and interpretative strategies rather than pooled quantitative deviation estimates. A systematic search of major biomedical databases was conducted to identify systematic reviews evaluating transfer accuracy in orthodontic indirect bonding. Data extraction was performed independently by two reviewers using a predefined methodological mapping framework, and methodological quality was assessed with AMSTAR-2. Four systematic reviews met the inclusion criteria. Across reviews, transfer accuracy was operationalized through heterogeneous linear and angular geometric deviation metrics derived from planned–achieved bracket position comparisons, without use of a standardized composite accuracy indicator. Nevertheless, substantial heterogeneity was found in outcome definitions, dimensional architectures, reference system selection, and analytical workflows, resulting in structurally non-equivalent representations of transfer accuracy and limiting cross-review comparability. Within the included systematic reviews, transfer accuracy functioned primarily as a workflow-dependent geometric measurement construct rather than as an outcome systematically operationalized within clinically validated frameworks. We recommend standardized construct definitions, mandatory reporting of reference systems and registration algorithms, routine uncertainty quantification, and harmonized dimensional frameworks as essential steps toward valid evidence synthesis, reproducible digital orthodontic workflows, and clinically interpretable transfer accuracy measurement.

## 1. Introduction

Accurate bracket placement is a critical determinant of orthodontic treatment control, as even small positional discrepancies can alter the expressed orthodontic prescription—that is, the intended torque, angulation, and rotational positioning encoded in bracket design [1]. Indirect bonding (IDB) was introduced to improve placement reproducibility while reducing chairside time and operator-dependent variability [2] by transferring brackets from a laboratory or digital setup to the clinical environment using positioning trays or transfer jigs [3,4]. Over the past decade, IDB has increasingly integrated with digital orthodontic workflows incorporating CAD/CAM planning, three-dimensional (3D) imaging, and additive manufacturing, expanding both technical capabilities and methodological complexity [5,6,7]. The integration of digital technologies into orthodontic workflows has expanded rapidly in recent years, enabling virtual treatment planning, CAD/CAM appliance fabrication, and digital indirect bonding systems [8]. Experimental investigations comparing different digital indirect bonding techniques have also reported measurable differences in transfer accuracy and chairside efficiency, supporting the clinical adoption of digitally assisted workflows [9].

Within this digital environment, transfer accuracy—representing the discrepancy between planned and achieved bracket positions—has emerged as a key performance metric for evaluating positional fidelity. In the present review, the term “transfer accuracy” refers to the overall outcome construct describing positional fidelity between planned and achieved bracket positions, whereas “transfer error” refers to the numerical linear or angular deviation metrics used to quantify that construct.

Digitally customized orthodontic systems based on CAD/CAM technologies have also been developed to facilitate bracket positioning and appliance fabrication within fully digital treatment workflows [10]. Systematic reviews typically quantify this outcome using linear and angular deviations derived from digital superimposition. However, the selection of coordinate axes, anatomical landmarks, dimensional frameworks, and measurement pipelines varies substantially across studies and evidence syntheses [5,6,11]. Consequently, positional fidelity is frequently interpreted as a geometric performance metric rather than as a clinically validated outcome directly linked to downstream treatment effects. This limitation reflects the absence of standardized validation frameworks connecting geometric deviation measurements to clinically interpretable treatment endpoints.

Measurement architecture itself represents a critical source of variability. Deviation values are inherently shaped by the selected reference system and analytical pipeline used to align planned and achieved bracket positions. For example, tooth-based and arch-based superimposition strategies generate distinct spatial reference frames, producing non-equivalent deviation measurements even when evaluating identical bracket positions [11,12]. Additional factors—including imaging modality, software-dependent registration algorithms, segmentation workflows, and coordinate system definitions—further influence measurement outputs. Reporting of measurement reliability and uncertainty remains inconsistent. Moreover, normative thresholds defining clinically acceptable deviations are widely applied despite limited empirical validation linking deviation magnitude to treatment outcomes [5,6,11]. This variability undermines bioengineering-driven optimizations in CAD/CAM workflows, where reproducible measurement frameworks are essential for automated analysis and digital system validation.

Related outcomes commonly synthesized in orthodontic bonding reviews—such as bracket failure rates, bond strength, and procedural efficiency—do not resolve these limitations. These outcomes assess distinct constructs and may coexist with substantial differences in positional measurement methodology [13,14,15]. As a result, systematic reviews aggregating positional accuracy across heterogeneous operational definitions risk synthesizing structurally non-equivalent constructs. This limits comparability and cumulative interpretation. These constraints arise primarily from differences in outcome operationalization and measurement architecture rather than from deficiencies in primary evidence. Examining how this outcome is defined, measured, and interpreted at the level of systematic evidence synthesis is therefore essential for understanding its validity and interpretability. From a measurement science perspective, outcomes defined primarily through geometric deviation metrics represent measurement constructs whose interpretability is inherently dependent on the methodological architecture used to generate them.

Despite rapid adoption of digital orthodontic technologies, systematic reviews evaluating positional fidelity remain fragmented by differences in conceptual definitions, measurement architectures, reference systems, and interpretative thresholds. Broader reviews of CAD/CAM-based orthodontic interventions have similarly highlighted the rapid expansion of digital workflows; they have also revealed substantial heterogeneity in technological applications and reporting practices [16]. This lack of standardized outcome operationalization not only limits cumulative evidence synthesis but also represents a critical barrier to technological advancement. Bioengineering innovations—including automated measurement pipelines, digital workflow optimization tools, and AI-driven superimposition algorithms—depend on clearly defined, reproducible, and interoperable measurement constructs. Without standardization, measurement outputs remain intrinsically workflow-dependent, constraining both clinical interpretability and technological scalability. Standardized measurement architectures are therefore required not only to enable valid evidence synthesis, but also to support calibration, benchmarking, and external validation of digital and AI-assisted orthodontic systems.

Unlike prior reviews primarily focused on clinical effectiveness or procedural efficiency, the present study conceptualizes transfer accuracy as a constructed measurement outcome and systematically maps the methodological architecture that governs its definition, quantification, and interpretability.

For the purposes of this review, transfer accuracy is defined as the geometric deviation between the digitally planned bracket position and the clinically achieved bracket position, quantified through linear (mm) and angular (°) measurements derived from three-dimensional digital superimposition, within a workflow-specific reference system.

Accordingly, this methodological umbrella review systematically analyzes how transfer accuracy is operationalized across systematic reviews.

## 2. Materials and Methods

### 2.1. Study Design

This study was conducted as a methodological umbrella review of published systematic reviews. The objective was to analyze how transfer accuracy in orthodontic indirect bonding is defined, measured, and interpreted at the level of evidence synthesis. Unlike effectiveness-focused umbrella reviews, the present analysis adopted an outcome-centered methodological framework, focusing on the structural components underlying outcome operationalization, including conceptual definitions, measurement architectures, dimensional frameworks, reference systems, and interpretative strategies. The review did not aim to compare clinical effectiveness or treatment performance. Instead, it sought to evaluate methodological characteristics affecting construct definition and cross-review comparability. Methodological umbrella reviews prioritize conceptual and measurement equivalence of the outcome construct across evidence syntheses rather than maximizing the number of included reviews, in order to ensure meaningful construct-level comparison.

### 2.2. Protocol of Registration

The protocol for this methodological umbrella review was registered in the International Prospective Register of Systematic Reviews (PROSPERO) (Registration ID: CRD420261322191). Although PROSPERO primarily hosts systematic reviews addressing clinical outcomes, protocol registration was undertaken to enhance methodological transparency and reproducibility.

### 2.3. Research Question

How is transfer accuracy in indirect bonding defined, measured, and reported across existing systematic reviews, and to what extent does methodological heterogeneity limit the comparability of findings?

### 2.4. Information Sources and Search Strategy

A systematic literature search was conducted in PubMed, Scopus, Web of Science, Embase, and the Cochrane Library. Database-specific search strategies were developed and adapted for each source.

### 2.5. Research Strategy

A comprehensive electronic search was carried out across the following databases:

PubMed, Scopus, Cochrane Library, Embase, and Web of Science.

The database search was completed in 16 February 2026. No restrictions regarding publication year or language were applied. Because transfer accuracy is typically reported within systematic reviews addressing orthodontic bonding techniques rather than as a standalone indexed outcome term, the search strategy was structured around orthodontic bracket bonding terminology combined with systematic review filters to maximize sensitivity of review identification. The complete search strategy for each database is reported in Table 1.

### 2.6. Review Type and Methodological Framework

The research question was framed using the Population–Concept–Context (PCC) framework, which is appropriate for reviews aiming to analyze outcome definition, measurement, and reporting rather than clinical effectiveness [17]. Consistent with the objectives of a methodological umbrella review, the focus was placed on outcome operationalization across systematic reviews rather than on evidence breadth or effectiveness mapping.

**Population:** Orthodontic bonding procedures and experimental models.

**Concept:** Transfer accuracy as an outcome related to the positional discrepancy between planned and bonded bracket positions.

**Context:** Systematic reviews synthesizing evidence on orthodontic bonding techniques.

The review was therefore designed to map methodological characteristics associated with transfer accuracy, including definitions, measurement models, dimensional approaches, reference systems, technological tools, and reporting practices, rather than to evaluate clinical outcomes or therapeutic effectiveness.

### 2.7. Eligibility Criteria

Eligibility criteria were defined to prioritize methodological relevance and outcome operationalization over clinical effectiveness synthesis.

#### 2.7.1. Inclusion Criteria

Systematic reviews were eligible for inclusion if they met all of the following criteria:

i. Reported a systematic search strategy with predefined eligibility criteria.
ii. Included studies on orthodontic indirect bonding of brackets.
iii. Addressed transfer accuracy as an outcome related to bracket positioning, defined or operationalized through planned–achieved position comparison.
iv. Reported or described at least one methodological component relevant to the assessment of transfer accuracy, including outcome definition, dimensional framework (2d/3d), reference system, measurement approach, analytical pipeline, or reporting strategy.

Systematic reviews including both direct and indirect bonding techniques were considered eligible only when transfer accuracy outcomes specific to indirect bonding could be clearly identified and methodologically characterized.

Reviews in which transfer accuracy was not explicitly defined were not excluded a priori, as the absence of a formal definition was considered methodologically informative for the purposes of outcome mapping.

#### 2.7.2. Exclusion Criteria

Articles were excluded if they were not systematic reviews designed to synthesize transfer accuracy outcomes using predefined analytical and interpretative methodologies (e.g., narrative reviews, expert opinions, editorials, letters, scoping reviews, or umbrella reviews).

Scoping reviews were excluded because their primary objective is typically evidence mapping rather than construct-level methodological synthesis, which was the focus of the present umbrella review.

### 2.8. Reporting Framework

The review was conducted and reported in accordance with the Preferred Reporting Items for Systematic Reviews and Meta-Analyses (PRISMA 2020) statement [18]. CONSORT and COSMIN frameworks were used exclusively as conceptual references to support structured interpretation of outcome definition, measurement validity, and interpretability, without performing formal checklist-based compliance assessment [19,20]. Although COSMIN was not applied as a formal checklist-based or taxonomic instrument, its conceptual domains informed the analytical structure of this review. Specifically, content validity considerations informed analysis of outcome definition and construct operationalization; structural validity considerations informed evaluation of dimensional frameworks, reference systems, and measurement architecture equivalence; and reliability considerations informed assessment of reproducibility and measurement uncertainty reporting across reviews. This application was conceptual and interpretative rather than checklist-based, and no formal COSMIN scoring or semantic taxonomy analysis was performed.

### 2.9. Data Extraction and Analytical Framework

Data extraction was independently performed by two reviewers (E.L.; A.V.) using a predefined methodological mapping framework specifically designed to capture the structural and methodological components underlying the definition, measurement, and interpretative architecture of transfer accuracy across systematic reviews. Inter-reviewer agreement was assessed using Cohen’s kappa coefficient, indicating almost perfect agreement (95% CI) for Cohen’s kappa Discrepancies were resolved through discussion and consensus.

Consistent with the methodological objective of this umbrella review, data extraction focused on outcome construction and measurement architecture rather than clinical effectiveness or pooled quantitative estimates. Extracted variables were predefined to enable structured construct-level analysis and methodological comparison across systematic reviews.

The following analytical domains were extracted:

i. Conceptual and operational definitions of transfer accuracy.
ii. Measurement constructs, including linear and angular deviation metrics and use of composite indices.
iii. Dimensional measurement frameworks (2d, 3d, or mixed approaches).
iv. Spatial reference systems used for positional superimposition and deviation quantification.
v. Measurement technologies, analytical software environments, and workflow-dependent pipelines.
vi. Reporting of measurement reliability, reproducibility, and uncertainty.
vii. Clinical interpretability frameworks including use and validation of clinical acceptability thresholds.
viii. Outcome synthesis strategies and handling of methodological heterogeneity.
ix. Methodological appraisal characteristics related to outcome validity and interpretability.
x. Interpretative framing of transfer accuracy as a geometric measurement construct or clinically validated clinical outcome.

The predefined data extraction framework and coding structure used for construct-level methodological mapping are provided in Supplementary Table S2.

### 2.10. Methodological Appraisal and Quality Assessment

The methodological quality of the included systematic reviews was assessed using the AMSTAR-2 [21] (A Measurement Tool to Assess Systematic Reviews 2) instrument. AMSTAR-2 evaluates key methodological domains of systematic reviews, including protocol registration, the literature search strategy, risk-of-bias assessment, and methods used for evidence synthesis.

In this methodological umbrella review, AMSTAR-2 was used exclusively to characterize the general methodological rigor and reporting completeness of the included systematic reviews rather than to evaluate the validity of the transfer accuracy measurement construct itself.

The appraisal was performed independently by two reviewers (E.L.; A.V.), and discrepancies were resolved through discussion and consensus.

## 3. Results

### 3.1. Study Selection

**Figure 1:**
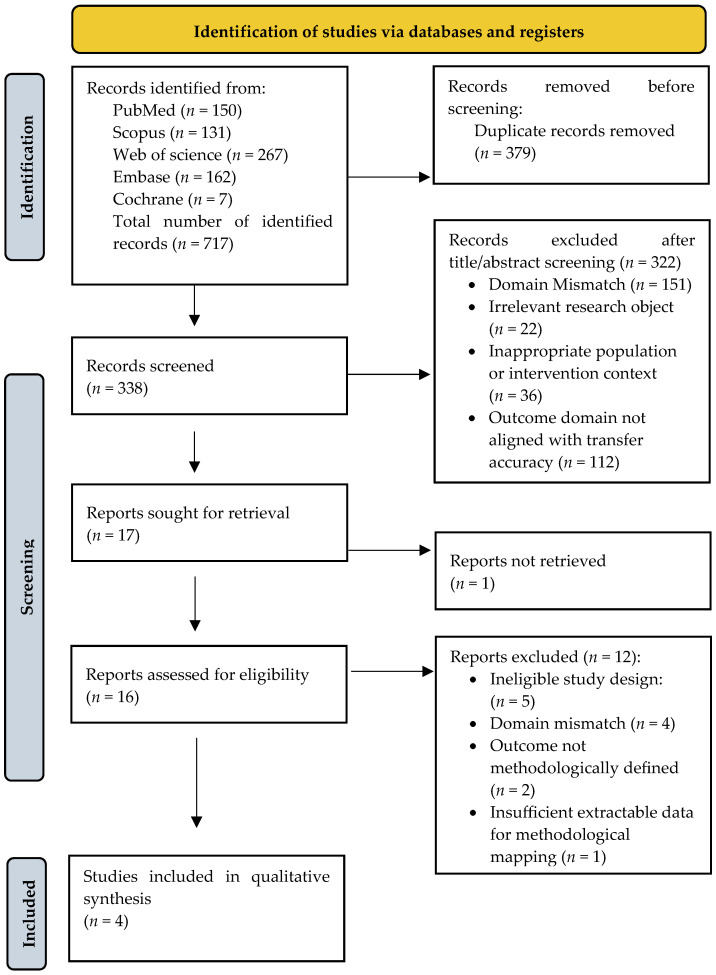
PRISMA flow diagram of study selection process. Records were identified through systematic searches of five electronic databases (PubMed, Scopus, Web of Science, Embase, Cochrane Library). Deduplication was performed prior to title/abstract screening. Full-text eligibility assessment was conducted independently by two reviewers. Abbreviations: *n* = number of records or reports.

The database search identified 717 records. After removal of 379 duplicates, 338 records underwent title and abstract screening. Seventeen reports were sought for retrieval, and 16 full-text articles were assessed for eligibility. Of these, 4 systematic reviews met the inclusion criteria and were included in the qualitative synthesis (Figure 1).

The exclusion of the remaining 12 full-text articles was primarily due to ineligible study design (narrative reviews, primary studies, or reviews not addressing transfer accuracy as a defined measurement outcome) or lack of extractable methodological information regarding outcome definition and measurement architecture.

Figure 1 presents the PRISMA flow diagram of the study selection process.

### 3.2. Characteristics of Included Studies

The four included systematic reviews were published between 2022 and 2024 and evaluated transfer accuracy in orthodontic indirect bonding, primarily within digital and CAD/CAM-based workflows. Across reviews, transfer accuracy was consistently defined as the positional discrepancy between planned and achieved bracket positions, quantified using linear and angular deviations derived from three-dimensional digital superimposition methods.

All reviews synthesized heterogeneous primary evidence, including in vitro, in vivo, ex vivo, or mixed study designs, with the number of included primary studies ranging from 9 to 16. Transfer accuracy was operationalized using geometric deviation metrics in linear (mesiodistal, buccolingual, vertical) and angular (angulation, torque, rotation) dimensions, reflecting the agreement between planned and transferred bracket positions.

Across reviews, transfer accuracy was treated as a geometric measurement outcome describing the technical fidelity of bracket transfer rather than as a clinically validated endpoint linked to treatment outcomes.

Key characteristics of the included systematic reviews are summarized in Table 2.

### 3.3. Methodological Quality of Included Systematic Reviews

The methodological quality of the included systematic reviews was assessed using the AMSTAR-2 instrument. Overall, the reviews demonstrated generally acceptable methodological rigor across most domains, particularly regarding research question formulation, the literature search strategy, duplicate study selection and data extraction procedures, and risk-of-bias assessment. However, several methodological limitations were observed, most notably incomplete reporting of excluded studies and absence of funding source reporting for primary studies. A detailed domain-level appraisal of each included systematic review is presented in Table 3.

### 3.4. Overlap and Structural Characteristics of Underlying Primary Evidence

Across the four included systematic reviews, 53 primary study occurrences were identified, corresponding to 26 unique primary studies, indicating substantial redundancy in the underlying evidence base.

Primary studies consisted predominantly of in vitro investigations, with fewer in vivo clinical studies. Transfer accuracy was consistently assessed using geometric deviation metrics, expressed as linear (mm) and angular (degrees) discrepancies between planned and achieved bracket positions derived from digital superimposition.

No systematic review reported clinically validated outcome measures, functional orthodontic endpoints, or patient-reported outcomes. Transfer accuracy was uniformly operationalized as a geometric measurement construct.

The Corrected Covered Area (CCA) was 0.35, the CCA was calculated according to the method proposed by Pieper et al., using the formula:

CCA = (N − r)/(rc − r)

where N represents the total number of primary study occurrences across reviews, r the number of unique primary studies, and c the number of included systematic reviews. Overlap analysis and CCA calculations were performed using manually verified extraction matrices and standard spreadsheet calculations.

This confirms that multiple reviews synthesized largely overlapping primary evidence, and that observed differences primarily reflect variation in measurement frameworks and methodological interpretation rather than differences in the underlying studies [22].

The overlap structure is presented in Table 4.

The full primary study-level overlap matrix, listing individual study inclusion across all four systematic reviews, is provided as Supplementary Table S1.

### 3.5. Methodological Architecture and Outcome Construction of Transfer Accuracy

Transfer accuracy was operationalized across systematic reviews using heterogeneous definitional models, measurement constructs, dimensional frameworks, reference systems, and interpretative approaches. While some reviews provided formal operational definitions, others relied on implicit or proxy measurement constructs derived from geometric deviation metrics. Across all reviews, transfer accuracy was consistently interpreted as a technical geometric construct governed by normative measurement thresholds rather than as a clinically validated outcome.

A structured methodological mapping of outcome construction domains across included systematic reviews is presented in Table 5.

To render the claim of structural non-equivalence concrete and reproducible, Table 6 presents a compact taxonomy classifying the measurement architectures of primary studies included across systematic reviews into four distinct classes: tooth-based 3D, arch-based 3D, tooth-based 2D, and arch-based 2D. A fifth category—Mixed/Unreported—captures primary studies in which the reference system or dimensional framework was not explicitly stated or varied across sub-analyses. As shown, all four systematic reviews draw simultaneously on primary studies belonging to multiple architecture classes, frequently synthesizing tooth-based and arch-based measurements, or 2D and 3D frameworks, under the unified outcome label of transfer accuracy. Because each architecture class generates geometrically non-equivalent deviation values for identical physical bracket positions, this co-occurrence of structurally distinct measurement paradigms within the same quantitative synthesis represents the primary mechanism underlying the fragmentation of reported findings identified in the present umbrella review [5,11,12].

#### 3.5.1. Definition of Transfer Accuracy

Across included systematic reviews, transfer accuracy lacked a standardized conceptual definition and was variably operationalized through geometric deviation metrics and workflow-dependent analytical choices, including reference system selection [5,11]. Sabbagh et al. explicitly defined transfer accuracy as the spatial discrepancy between planned and achieved bracket positions quantified by linear and angular deviations [11]. Palone et al. similarly operationalized transfer accuracy but demonstrated that reported values varied depending on the superimposition reference system [12]. In contrast, Bakdach & Hadad and Campobasso et al. did not provide formal conceptual definitions and instead inferred transfer accuracy from reported geometric deviation measures [5,6]. Overall, transfer accuracy emerged as a measurement-defined construct rather than a clinically validated endpoint, and none of the included systematic reviews linked it to treatment effectiveness, functional outcomes, or patient-centered measures [5,12].

#### 3.5.2. Measurement Approaches

All included systematic reviews operationalized transfer accuracy using separate linear (mm) and angular (°) deviation measurements derived from comparison of planned and achieved bracket positions [5,11]. Linear deviations quantified translational discrepancies along spatial axes, whereas angular deviations quantified rotational discrepancies, including torque, angulation, and rotation [6,11]. None of the reviews reported the use of a validated composite accuracy index integrating linear and angular components into a single outcome measure. Instead, linear and angular deviations were consistently reported and analyzed as separate geometric parameters [5,12].

#### 3.5.3. Dimensional Frameworks

Three-dimensional (3D) measurement approaches predominated across the included systematic reviews, consistent with the digital workflows used in indirect bonding [6,12]. However, two-dimensional (2D) and mixed 2D–3D measurement approaches were also represented among the synthesized primary studies [5,6]. Linear and angular deviations were derived using dimensional frameworks corresponding to the underlying measurement modality. Despite this heterogeneity, measurements obtained from 2D, 3D, and mixed frameworks were synthesized and reported under the same outcome construct of transfer accuracy [5,11].

#### 3.5.4. Reference Systems

Reference system selection varied across included systematic reviews and synthesized primary studies, with both arch-based and tooth-based superimposition strategies reported [5,11]. Palone et al. reported that deviation magnitudes differed depending on the selected superimposition reference system, including arch-based and tooth-based approaches [12]. Sabbagh et al. and Bakdach & Hadad included primary studies using heterogeneous reference systems without a standardized reference framework [6,11]. Transfer accuracy measurements were therefore derived from different reference system configurations across studies [5,12].

#### 3.5.5. Measurement Tools and Technologies

Transfer accuracy assessment was conducted using heterogeneous technological environments, including optical scanners, CBCT imaging, digitized physical models, and CAD/CAM-based analysis platforms [5,6]. Software environments included proprietary CAD/CAM software, commercial orthodontic software, and custom digital measurement workflows [11,12]. None of the included systematic reviews identified or reported the use of a standardized measurement pipeline across primary studies [5,11]. Transfer accuracy measurements were derived using study-specific technological and software configurations.

#### 3.5.6. Measurement Reliability and Error

None of the included systematic reviews reported formal assessment of measurement reliability or quantitative evaluation of measurement uncertainty [5,11]. Sabbagh et al. and Bakdach & Hadad acknowledged potential sources of measurement variability, including scanner resolution, transfer tray deformation, and registration procedures [6,11]. However, none of the reviews reported quantitative modeling, propagation analysis, or uncertainty-adjusted accuracy estimates. Transfer accuracy values were reported as geometric deviation measurements without formal reliability or uncertainty quantification [5,12].

#### 3.5.7. Thresholds of Clinical Relevance

Clinical acceptability thresholds were referenced across multiple systematic reviews, most commonly 0.5 mm for linear deviations and 2° for angular deviations [5,11,12]. These thresholds were reported in relation to established orthodontic reference standards, including American Board of Orthodontics criteria [5,12]. None of the included systematic reviews reported empirical validation of these thresholds through direct association with clinical outcome measures, such as treatment duration, occlusal function, or bracket repositioning frequency [5,11].

#### 3.5.8. Outcome Synthesis Strategies

All included systematic reviews employed both quantitative and narrative synthesis approaches to analyze transfer accuracy outcomes [5,11]. Quantitative synthesis aggregated linear and angular deviation measurements derived from primary studies using different dimensional frameworks, reference systems, and measurement technologies [11,12]. Narrative synthesis was also used to describe methodological characteristics and sources of heterogeneity across included studies [5,11].

#### 3.5.9. Methodological Appraisal Patterns

Quality appraisal methods reported in the included systematic reviews addressed general aspects of systematic review methodology, including search strategy, study selection, and risk of bias assessment [5,11]. None of the reviews reported formal evaluation of outcome-specific methodological properties, such as construct validity, measurement reliability, or interpretability of transfer accuracy measurements. [5,12]. Methodological appraisal focused on study-level quality assessment rather than outcome-specific measurement characteristics [6,11].

#### 3.5.10. Interpretative Framing

Across all included systematic reviews, transfer accuracy was interpreted as a technical performance metric derived from geometric deviation measurements between planned and achieved bracket positions [5,11]. Reported outcomes were expressed as linear and angular deviations, typically evaluated in relation to previously reported technical reference thresholds [5,12].

None of the reviews interpreted transfer accuracy using validated clinical outcome measures or direct associations with treatment effectiveness, indicating that the construct was assessed primarily within a technical measurement framework rather than as a clinically validated outcome domain [5,11].

#### 3.5.11. Results Synthesis

Across the included systematic reviews, transfer accuracy was consistently quantified using geometric deviation metrics, but substantial methodological heterogeneity was observed in its definition and measurement. Variability emerged in dimensional frameworks, reference systems, measurement technologies, and analytical pipelines, resulting in non-equivalent representations of transfer accuracy across reviews [5,11].

Consequently, transfer accuracy currently functions as a workflow-dependent measurement construct rather than a construct systematically operationalized and interpreted within clinically validated outcome frameworks across the included systematic reviews, which limits cross-review comparability and constrains cumulative interpretation of the available evidence [11,12].

## 4. Discussion

### 4.1. Principal Findings and Contribution

This methodological umbrella review demonstrates that transfer accuracy in orthodontic indirect bonding is consistently operationalized as a geometric deviation metric derived from planned–achieved bracket position comparisons, rather than as a clinically validated outcome domain [5,11].

Across included systematic reviews, heterogeneity originates primarily at the level of outcome operationalization rather than solely at the level of effect estimates. Specifically, differences in definitional criteria, dimensional frameworks, reference systems, measurement pipelines, and interpretative thresholds generate structurally non-equivalent measurement outputs reported under the same nominal outcome label of “transfer accuracy.”

Importantly, this observation reflects a characteristic of the current evidence synthesis landscape rather than a methodological limitation of the present umbrella review. The absence of clinically validated outcome frameworks represents a property of the existing literature itself, as none of the included systematic reviews operationalized transfer accuracy using validated clinical outcome constructs.

An important implication of these findings is that variability in reported transfer accuracy values primarily reflects differences in measurement architecture rather than differences in the underlying primary evidence base. This interpretation is supported by the substantial overlap of primary studies across systematic reviews (CCA = 0.35), indicating that divergent findings frequently arise from methodological differences in outcome definition, measurement, and interpretation rather than from differences in included primary data.

This finding is consistent with prior orthodontic literature describing variability in bracket positioning accuracy depending on measurement methodology, digital workflow characteristics, and superimposition strategies, even when identical bonding protocols are evaluated [4,23,24].

Accordingly, the principal contribution of this umbrella review is to demonstrate that transfer accuracy currently appears to function primarily as a measurement-dependent technical performance parameter rather than as a standardized and clinically validated outcome construct.

Importantly, this conclusion should be interpreted epistemically and at the level of evidence synthesis design. Specifically, the included systematic reviews were not designed to evaluate associations between transfer accuracy deviation magnitude and clinical treatment outcomes. Consequently, the present findings identify the absence of clinical outcome operationalization within existing systematic review frameworks rather than definitive evidence that transfer accuracy lacks clinical relevance or validity as a construct.

### 4.2. Positioning Within the Broader Orthodontic Literature and Construct Evolution

Historically, orthodontic systematic reviews evaluating indirect bonding have primarily focused on clinical performance endpoints such as bond failure rates, treatment efficiency, or procedural workflow efficiency, rather than on the methodological properties underlying positional accuracy measurement. Previous umbrella-level evidence synthesis in this field has also focused predominantly on effectiveness and efficiency outcomes rather than on how transfer accuracy is conceptually defined, measured, and reported [25]. Earlier literature commonly assessed bracket positioning using manual gauges, two-dimensional measurements, or radiographic estimation, where positional accuracy was interpreted in relation to clinical acceptability thresholds rather than as a quantitatively defined geometric deviation construct [26]. It should be acknowledged, however, that digital orthodontic workflows have evolved rapidly since 2018–2020, with substantial advances in intraoral scanner accuracy, CAD/CAM tray fabrication, automated superimposition algorithms, and AI-assisted bracket positioning, such that measurement paradigms and achievable accuracy levels reported in pre-2020 literature may not fully reflect the current technological landscape [8,9,10].

This interpretation is consistent with broader orthodontic literature describing bracket positioning error as a multifactorial construct influenced by operator technique, transfer tray design, tooth morphology, bonding protocol variability, and material characteristics. Previous literature has emphasized that bracket positioning errors arise from multiple biological, procedural, and technical sources, highlighting that positional discrepancy represents a context-dependent construct influenced by workflow-specific factors [27,28]. This conceptual framework supports the interpretation that positional deviation measurements cannot be assumed to represent a universally standardized outcome construct independent of methodological context.

The introduction of digital orthodontic workflows incorporating CAD/CAM planning, optical scanning, and three-dimensional superimposition has fundamentally transformed the operationalization of positional accuracy. Transfer accuracy is now quantified through geometric comparison of planned and achieved bracket positions within digital coordinate systems. Studies evaluating digital orthodontic setups have shown that the accuracy of virtual treatment planning depends on the reliability of digital models, segmentation procedures, and superimposition techniques [29]. However, this transition has introduced additional methodological dependencies, including reference system selection, coordinate system definition, superimposition algorithms, and software-dependent registration procedures, all of which directly influence measurement outputs [11,12]. Prior narrative and systematic literature has similarly acknowledged that digital workflows introduce measurement variability associated with acquisition accuracy, segmentation procedures, and alignment methodologies, further emphasizing the methodological complexity underlying positional accuracy assessment [4,23,30].

The limited number of eligible systematic reviews identified in this umbrella review (*n* = 4) is consistent with the relatively recent adoption of digital superimposition-based measurement paradigms within orthodontic research. Earlier systematic and narrative reviews frequently employed measurement approaches lacking methodological equivalence with contemporary three-dimensional planned–achieved positional analysis. Inclusion of such studies would have introduced structural heterogeneity unrelated to the outcome construct evaluated in the present review, thereby limiting the validity of construct-level methodological synthesis.

Prioritizing methodological comparability and measurement equivalence over sample size is consistent with the objectives of methodological umbrella reviews focusing on outcome operationalization. Restricting inclusion to systematic reviews employing comparable geometric deviation frameworks enables accurate evaluation of how transfer accuracy is defined, measured, and interpreted within the contemporary digital orthodontic evidence base. Accordingly, the limited number of included systematic reviews reflects the current methodological boundaries of synthesized evidence evaluating transfer accuracy as a geometric measurement construct derived from digital indirect bonding workflows.

### 4.3. Methodological Interpretation: Why Findings Fragment Despite Shared Evidence

**Figure 2:**
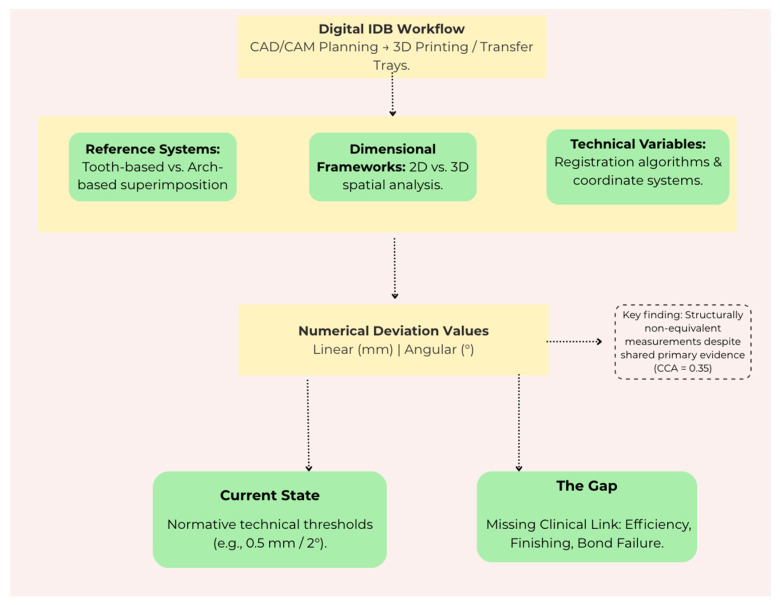
Hierarchical model illustrating how methodological factors influence the operationalization and interpretability of transfer accuracy in digital indirect bonding workflows. Each level represents a distinct tier of methodological influence, from foundational measurement inputs to clinical applicability. Arrows indicate the direction of propagation of methodological variability across levels. Abbreviations: CAD/CAM = computer-aided design/computer-aided manufacturing; 2D = two-dimensional; 3D = three-dimensional; AI = artificial intelligence.

The observed fragmentation in transfer accuracy findings across systematic reviews appears to arise primarily from differences in measurement architecture rather than from differences in the underlying primary evidence base. This hierarchical mechanism is illustrated in Figure 2, which conceptualizes how methodological parameters governing measurement construction—including reference system selection, dimensional framework, coordinate modeling, and technical implementation—directly influence numerical deviation outputs. Under these conditions, transfer accuracy values represent workflow-dependent geometric constructs whose interpretability is intrinsically shaped by measurement architecture rather than solely by the physical positioning of brackets. A hierarchical synthesis of these sources of methodological heterogeneity across the included systematic reviews is further detailed in Table 7.

Reference system selection represents a critical determinant of measurement output. Tooth-based superimposition isolates positional discrepancies at the individual tooth level, preserving localized deviations between planned and achieved bracket positions. In contrast, arch-based superimposition distributes positional discrepancies across the entire dental arch, potentially attenuating localized deviations through global alignment effects [5,12]. Consequently, identical physical bracket positions may yield different reported deviation magnitudes depending on the reference system employed. This dependency indicates that numerical deviation values cannot be interpreted independently of the underlying spatial reference framework used for superimposition and measurement [28].

Dimensional measurement framework selection further contributes to structural non-equivalence across systematic reviews. Two-dimensional measurement approaches quantify projected positional discrepancies within planar reference systems, whereas three-dimensional analysis captures volumetric spatial deviation across all translational and rotational axes [24]. Differences in measurement architecture and analytical frameworks are well known to influence reproducibility and reliability in orthodontic measurements [31]. These fundamentally different measurement paradigms represent distinct geometric constructs and cannot be assumed to produce interchangeable or directly comparable deviation values. Despite this, systematic reviews frequently synthesize findings derived from heterogeneous dimensional frameworks under the unified outcome label of transfer accuracy, thereby introducing structural heterogeneity at the level of outcome definition.

Technical implementation factors, including software-dependent registration algorithms, segmentation procedures, coordinate system definitions, and model alignment workflows, introduce additional layers of measurement variability [32]. These methodological parameters directly influence how digital models are aligned and how positional discrepancies are computed, yet they are inconsistently reported and rarely incorporated into uncertainty analysis or reliability modeling. As a result, measurement pipelines themselves function as outcome-defining components rather than neutral analytical tools.

It should be noted that the present umbrella review characterizes reference-system-dependent and registration-method-dependent variability qualitatively rather than quantitatively. Formal sensitivity analysis and computational error-propagation modeling would require access to raw primary-study measurement datasets, which were unavailable within the included systematic reviews.

Accordingly, the present findings identify the absence of standardized sensitivity and uncertainty modeling as a major methodological gap in the current literature. Future engineering-oriented investigations should quantify the range of deviation variability attributable to reference-system selection and model error accumulation across measurement pipeline stages, including acquisition, segmentation, registration, superimposition, and coordinate decomposition workflows.

These dependencies operate within a hierarchical structure, as summarized in Table 6, where methodological heterogeneity at the level of measurement architecture propagates upward to influence outcome interpretation and clinical applicability. At the methodological level, heterogeneous reference systems, dimensional frameworks, and analytical pipelines generate structurally distinct geometric representations of positional accuracy. At the mechanistic level, workflow-dependent measurement pipelines introduce systematic variability in reported deviation values. At the epistemic level, these methodological differences limit the interpretability and comparability of reported transfer accuracy measurements across systematic reviews. At the clinical level, these constraints restrict the extent to which transfer accuracy can be interpreted as a universally standardized or clinically validated outcome construct.

Overlap analysis further supports this interpretation. Despite substantial overlap in primary study inclusion across systematic reviews (CCA = 0.35), reported transfer accuracy findings remain fragmented. This pattern indicates that variability in reported accuracy values arises primarily from methodological differences in outcome operationalization rather than from differences in the underlying primary evidence base. Under these conditions, transfer accuracy is most appropriately interpreted as a measurement-dependent construct whose numerical representation reflects methodological architecture rather than a universally equivalent clinical outcome measure.

#### Methodological Quality and Synthesis Robustness

Methodological quality, assessed using AMSTAR-2, ranged from low to moderate across the included systematic reviews. These limitations primarily reflected incomplete protocol registration, incomplete reporting of excluded studies, and inconsistent integration of risk-of-bias assessments into result interpretation [5,11]. Such limitations indicate variability in methodological rigor and reporting completeness across systematic reviews synthesizing transfer accuracy outcomes. AMSTAR-2 was used to characterize general methodological rigor and reporting completeness of the included systematic reviews rather than to evaluate outcome-specific measurement validity.

Because the present umbrella review was designed to evaluate outcome operationalization and measurement architecture rather than to estimate treatment effects or comparative effectiveness, these methodological limitations do not directly affect the validity of construct-level interpretation. Instead, they provide important contextual insight into how transfer accuracy has been historically evaluated within systematic evidence synthesis. Specifically, the observed methodological patterns suggest that transfer accuracy has predominantly been operationalized as a geometric measurement construct, without systematic evaluation of outcome validity, measurement reliability, or clinical interpretability within standardized outcome frameworks.

Overlap analysis further supports the interpretation that fragmentation in reported findings arises primarily from methodological heterogeneity in outcome operationalization rather than from differences in the underlying primary evidence base. Despite substantial overlap in primary study inclusion across systematic reviews (CCA = 0.35), reported transfer accuracy findings remained structurally heterogeneous [5,12]. This pattern indicates that differences in reference system selection, dimensional framework, and measurement architecture represent primary determinants of variability in reported accuracy values.

Under these conditions, fragmentation in systematic review findings is most appropriately interpreted as a consequence of methodological non-equivalence in outcome definition and measurement architecture rather than as a reflection of inconsistency in primary evidence or deficiencies in the literature coverage. This interpretation reinforces the conclusion that transfer accuracy currently functions as a measurement-dependent construct whose numerical representation is intrinsically shaped by methodological implementation.

AMSTAR-2 was used to characterize general methodological rigor and reporting completeness of the included systematic reviews rather than to evaluate outcome-specific measurement validity or construct validity of transfer accuracy itself.

### 4.4. Clinical Interpretability and the Problem of Normative Thresholds

Interpretation of transfer accuracy values frequently relies on normative technical thresholds, most commonly linear deviations of approximately 0.5 mm and angular deviations of 2° [5,11,12]. These thresholds originate from historical orthodontic standards, including Andrews’ straight-wire prescription framework and American Board of Orthodontics (ABO) evaluation criteria, where acceptable bracket positioning tolerances were defined based on clinical conventions and manual measurement capabilities.

It is important to distinguish between two structurally different categories of threshold: convention-based thresholds and empirically derived thresholds. Convention-based thresholds—such as the 0.5 mm/2° values consistently referenced across the included systematic reviews [5,11,12]—were established through expert consensus and historical clinical practice, predating contemporary three-dimensional digital superimposition workflows. They represent agreed-upon technical reference values rather than thresholds derived from direct empirical observation of clinical consequences. Empirically derived thresholds, by contrast, would be grounded in prospective or retrospective clinical data demonstrating that positional deviations above a specific magnitude are associated with measurable adverse outcomes—such as increased bracket repositioning frequency, reduced finishing accuracy, prolonged treatment duration, or suboptimal occlusal results. No such empirically derived threshold has been established within the systematic review literature analyzed in the present umbrella review, and none of the included reviews reported a direct association between deviation magnitude and any clinically meaningful endpoint [5,11,12].

Furthermore, because deviation magnitude is intrinsically dependent on measurement architecture—including reference system selection, coordinate system definition, and software-dependent alignment procedures—identical numerical thresholds may correspond to structurally non-equivalent spatial discrepancies across workflows [5,12]. Previous orthodontic literature has similarly emphasized that clinically acceptable positional tolerances may vary depending on measurement methodology and technological context [4].

To establish empirically grounded thresholds, future research should consider prospective clinical study designs in which transfer accuracy is measured using standardized three-dimensional superimposition protocols at the time of bracket placement, and patients are subsequently followed through active orthodontic treatment to documented endpoints. Specifically, a prospective cohort study could enroll patients undergoing digital indirect bonding across a range of initial deviation magnitudes (e.g., stratified into ≤0.25 mm, 0.25–0.5 mm, 0.5–1.0 mm, and >1.0 mm linear deviation bands, with corresponding angular strata), and record the following downstream clinical outcomes: (i) bracket repositioning rate and timing; (ii) finishing accuracy evaluated using a validated index such as the ABO Objective Grading System (OGS); (iii) total active treatment duration; and (iv) number of wire adjustments or auxiliary mechanics attributable to positional correction. Regression modeling of deviation magnitude against these endpoints—controlling for tooth type, arch position, tray material, and operator experience—would allow the identification of deviation thresholds above which clinically meaningful deterioration in outcomes is reliably observed. Such a design would provide the empirical foundation currently absent from the literature [5,11] and transform transfer accuracy from a convention-referenced technical metric into a clinically interpretable and validated outcome measure.

Under these conditions, threshold-based interpretation is most appropriately understood as a measurement-dependent classification framework rather than a universally standardized clinical decision criterion [5,12]. This interpretation does not diminish the clinical relevance of bracket positioning accuracy, but underscores that the clinical interpretability of transfer accuracy measurements remains intrinsically linked to methodological context, measurement architecture, and—critically—the empirical validation of the thresholds used to classify positional fidelity.

### 4.5. Implications for Future Research and Evidence Synthesis

The present findings underscore the need for methodological standardization in defining, measuring, and reporting transfer accuracy in digital indirect bonding workflows. Priority areas include explicit operational definitions, standardized reference system reporting, harmonized coordinate specifications, and routine quantification of measurement reliability and uncertainty [11,12].

At the primary study level, future investigations should explicitly characterize measurement architecture—including reference systems, superimposition methodologies, coordinate frameworks, and analytical software environments—to enhance reproducibility and methodological transparency.

Future engineering-oriented investigations should also incorporate formal sensitivity analyses and uncertainty propagation modeling to quantify how reference-system selection, registration algorithms, and alignment workflows influence reported transfer accuracy measurements.

Empirical validation studies are also essential to establish robust relationships between geometric deviation metrics and clinically relevant outcomes, such as bracket repositioning, finishing accuracy, and treatment efficiency, thereby strengthening the clinical interpretability of positional accuracy measurements [4,23].

From a bioengineering and digital systems perspective, the absence of standardized measurement architectures represents a fundamental constraint on the development and validation of automated analytical tools. Emerging AI-driven superimposition algorithms, automated registration pipelines, and computational outcome evaluation systems require clearly defined and interoperable measurement constructs to ensure reproducibility, scalability, and clinical reliability. Without standardized reference frameworks and outcome definitions, algorithmic outputs remain intrinsically workflow-dependent, limiting both technological scalability and clinical integration.

At the evidence synthesis level, quantitative meta-analysis of transfer accuracy is most appropriate when measurement equivalence across studies can be reasonably established. Where measurement architectures differ substantially, structured methodological mapping approaches—as demonstrated in the present umbrella review—provide a more valid and interpretable synthesis framework than statistical aggregation alone.

Future systematic and umbrella reviews should therefore prioritize explicit evaluation of outcome operationalization and measurement architecture as core methodological domains, supporting the development of standardized, transparent, and clinically interpretable outcome frameworks in digital orthodontics.

#### Minimum Reporting Standards for Transfer Accuracy in Future Primary Studies

Building on the methodological gaps identified in the present umbrella review, the following minimal reporting items are proposed as a prescriptive framework for future primary studies evaluating transfer accuracy in digital indirect bonding workflows. These items address the principal sources of measurement variability and uncertainty identified across the included systematic reviews [5,11,12] and are intended to support reproducibility, methodological transparency, and cross-study comparability.

**(i) Scanner resolution and acquisition parameters.** Authors should report the type, model, and nominal resolution (in mm or μm) of the intraoral or laboratory scanner used for digital model acquisition, along with any post-acquisition processing steps (e.g., point cloud filtering, mesh decimation) that may affect geometric fidelity. Minimum reporting should include: scanner model, nominal accuracy as stated by the manufacturer, and scan protocol (e.g., number of scans, stitching method).

**(ii) Registration algorithm and superimposition methodology.** The algorithm used to align planned and achieved bracket positions should be explicitly described, including whether iterative closest point (ICP), surface-based, landmark-based, or hybrid registration was employed. The anatomical or geometric basis for alignment—tooth surface, palatal rugae, arch geometry, or other—should be stated. Software environment and version should be reported to enable methodological replication.

**(iii) Coordinate system definition.** The coordinate system used to decompose positional deviations into linear (mesiodistal, buccolingual, vertical) and angular (torque, angulation, rotation) components should be explicitly defined, including its origin, axis orientations, and whether it is tooth-referenced or arch-referenced. Authors should report whether the coordinate system was defined manually, semi-automatically, or automatically, and by which software module.

**(iv) Intra- and inter-rater reliability.** Measurement reliability should be formally assessed and reported for all outcome measurements. Minimum reporting should include: intraclass correlation coefficient (ICC) with 95% confidence intervals for continuous deviation measures, calculated from a representative subsample (recommended minimum: 10–15 cases, measured on two separate occasions by the same rater and by a second independent rater). The ICC model type (two-way mixed, absolute agreement) should be specified.

**(v) Measurement uncertainty quantification.** Authors should report a quantitative estimate of measurement uncertainty associated with the full measurement pipeline, from acquisition to deviation calculation. Acceptable formats include: repeatability standard deviation (SD) derived from repeated measurements of the same specimen under identical conditions; limits of agreement from Bland–Altman analysis; or total measurement uncertainty expressed as an expanded uncertainty interval (k = 2, 95% coverage). Where full uncertainty propagation is not feasible, authors should at minimum report the technical error of measurement (TEM) and relative TEM (%TEM) for each outcome variable.

Adoption of these minimal reporting items does not presuppose methodological uniformity across studies, but ensures that the measurement architecture underlying reported transfer accuracy values is sufficiently transparent to allow cross-study comparability, pooled analysis, and critical appraisal. Future systematic reviews and meta-analyses should consider these items as eligibility or quality criteria when evaluating primary study methodology [11,12].

### 4.6. Strengths

This methodological umbrella review presents several methodological and analytical strengths.

First, its construct-centered analytical framework enabled systematic examination of transfer accuracy as an outcome construct, rather than as a clinical effectiveness endpoint. This approach facilitated identification of synthesis-level fragmentation arising from differences in outcome definition, measurement architecture, dimensional frameworks, reference systems, and interpretative models across systematic reviews [5,11].

Second, the review applied a predefined methodological mapping framework aligned with outcome construct analysis, enabling structured evaluation of conceptual definitions, measurement constructs, dimensional frameworks, reference systems, measurement pipelines, interpretative thresholds, and synthesis strategies across systematic reviews [5,11,12]. This framework supported comprehensive characterization of how transfer accuracy is operationalized and interpreted at the level of systematic evidence synthesis.

The interpretative framework was informed by established methodological principles derived from outcome reporting (CONSORT) and measurement validity (COSMIN), applied as conceptual reference frameworks to support structured construct-level analysis and methodological interpretation.

Third, overlap analysis using the Corrected Covered Area (CCA) provided quantitative characterization of shared primary evidence across systematic reviews, supporting construct-level interpretation of synthesis heterogeneity [5,12].

Fourth, methodological appraisal using AMSTAR-2 enabled structured evaluation of methodological rigor and reporting completeness of the included systematic reviews, providing contextual support for interpretation of synthesis-level methodological variability [6,11].

Finally, protocol preregistration, duplicate study selection and data extraction procedures, and transparent methodological reporting supported reproducibility, transparency, and methodological rigor of the umbrella review process.

### 4.7. Limitations

This methodological umbrella review has several limitations.

First, the number of included systematic reviews was limited (*n* = 4). This limitation is consistent with the current state of systematic evidence specifically evaluating transfer accuracy using planned–achieved positional discrepancy frameworks within digital orthodontic workflows [5,8]. Restricting inclusion to systematic reviews employing comparable measurement paradigms supported construct-level methodological analysis while reducing structural heterogeneity arising from fundamentally different outcome definitions and measurement approaches.

Second, the methodological quality of the included systematic reviews, assessed using AMSTAR-2, ranged from low to moderate. These limitations primarily involved incomplete protocol registration, incomplete reporting of excluded studies, and inconsistent integration of risk-of-bias assessments into synthesis interpretation [5,8]. These methodological characteristics should be considered when interpreting synthesis-level findings, as variability in systematic review rigor may influence the completeness and transparency of reported outcome operationalization.

Third, the analysis was constrained by the reporting completeness of the included systematic reviews. Inconsistent disclosure of measurement pipelines, reference system selection, coordinate system definitions, and measurement reliability limited the granularity of methodological classification and synthesis-level comparison [12]. In addition, no formal sensitivity analysis or computational error-transfer modeling could be performed because the included systematic reviews and underlying primary studies inconsistently reported registration workflows, reference-system parameters, and uncertainty propagation methodologies. Consequently, the present umbrella review was limited to construct-level methodological mapping rather than quantitative metrological modeling. In addition, previous systematic reviews have evaluated the clinical efficiency of indirect bonding procedures rather than the methodological properties of positional accuracy measurement [33]. Finally, transfer accuracy measurement paradigms represent relatively recent developments within digital orthodontics, and the available systematic evidence remains methodologically heterogeneous [11,12]. As additional systematic reviews employing standardized measurement architectures, explicit outcome definitions, and improved reporting practices become available, future umbrella reviews may enable more refined construct-level methodological analysis and outcome framework standardization. The exclusion of primary studies was intentional and constitutes a defining methodological choice of the present umbrella review. Because the analytical objective was to examine how transfer accuracy is defined, measured, and interpreted at the level of systematic evidence synthesis—rather than to evaluate individual experimental measurements or estimate pooled effect sizes—restricting inclusion to systematic reviews was necessary to ensure construct-level methodological comparability. This choice, however, affects the generalizability of the findings in a specific and bounded way: conclusions drawn here pertain to the methodological architecture of transfer accuracy as operationalized within systematic review literature, and may not fully reflect the measurement practices of individual primary studies that were not captured or synthesized within the included reviews. In particular, more recent primary studies employing standardized measurement pipelines or novel superimposition methodologies may not be represented in the synthesized evidence base, given that their findings may not yet have been incorporated into published systematic reviews. Future umbrella reviews incorporating both systematic reviews and high-quality primary studies employing comparable measurement architectures may provide a more granular and comprehensive characterization of transfer accuracy operationalization across the full evidence base.

## 5. Conclusions

This methodological umbrella review demonstrates that transfer accuracy in orthodontic indirect bonding is currently operationalized within the systematic review literature as a geometric deviation construct derived from planned–achieved bracket position comparisons rather than as an outcome systematically operationalized and interpreted within clinically validated frameworks across the included systematic reviews.

Substantial heterogeneity in conceptual definitions, dimensional frameworks, reference systems, registration workflows, and measurement architectures results in structurally non-equivalent representations of transfer accuracy across systematic reviews, thereby limiting cross-review comparability despite partial overlap of the underlying primary evidence base.

Under these conditions, transfer accuracy is most appropriately interpreted as a workflow-dependent measurement construct whose numerical representation is intrinsically shaped by methodological implementation and measurement architecture. Importantly, this conclusion should be interpreted at the level of evidence synthesis design: the included systematic reviews were not designed to evaluate associations between transfer accuracy deviation magnitude and clinically meaningful treatment outcomes. Accordingly, the present findings identify a gap in current evidence synthesis frameworks rather than definitive evidence regarding the absence of clinical relevance of transfer accuracy as a construct.

Methodological harmonization—including standardized construct definitions, transparent reporting of measurement architecture, reproducibility frameworks, and empirical investigation of clinically meaningful deviation thresholds—is necessary to support valid evidence synthesis, reproducible digital orthodontic workflows, and improved clinical interpretability. This umbrella review provides a methodological foundation for future standardization of outcome definition, measurement, interpretation, and reporting of transfer accuracy within digital orthodontic research.

To translate this methodological framework into future research and evidence synthesis practice, the following priorities are proposed:

- **For researchers designing primary studies:** Explicitly report key components of measurement architecture underlying transfer accuracy assessment, including reference systems, dimensional frameworks, registration workflows, coordinate system definitions, and quantitative reliability estimates, as part of standardized methodological reporting.
- **For authors of systematic reviews and meta-analyses:** Quantitative synthesis of transfer accuracy outcomes should explicitly account for heterogeneity in dimensional frameworks, reference systems, and measurement architectures in order to avoid structurally non-equivalent comparisons and potentially misleading aggregation of deviation metrics.
- **For clinical researchers and trialists:** Prioritize prospective investigations evaluating associations between transfer accuracy deviation magnitude and clinically meaningful treatment outcomes within standardized measurement frameworks, with the aim of establishing empirically grounded acceptability thresholds.
- **For journal editors and peer reviewers:** Greater emphasis on transparent reporting of measurement architecture and reproducibility parameters may improve methodological consistency, cumulative evidence synthesis, and interpretability across digital orthodontic research.

## Figures and Tables

**Table 1:** Search Strategy for each database.

Database	Search Strategy
Pubmed	((“Orthodontic Brackets”[MeSH Terms] OR “bracket*”[Title/Abstract]) AND “bond*”[Title/Abstract] AND (“review”[Title/Abstract] OR “systematic review”[Publication Type] OR “systematic review”[Title/Abstract] OR “meta-analysis”[Publication Type] OR “meta-analysis”[Title/Abstract])) NOT (“letter”[Publication Type] OR “editorial”[Publication Type] OR “comment”[Publication Type])
Embase	((‘orthodontic bracket’/exp OR bracket*:ti,ab) AND (bond*:ti,ab) AND (review:ti,ab OR ‘systematic review’/it OR ‘systematic review’:ti,ab OR ‘meta analysis’/it OR ‘meta analysis’:ti,ab)) NOT (letter/it OR editorial/it)
Scopus	TITLE-ABS-KEY (bracket*) AND TITLE-ABS-KEY (bond*) AND TITLE-ABS-KEY (review OR “systematic review” OR “meta-analysis”) AND (LIMIT-TO (DOCTYPE, “re”))
Web of Science	TS = (bracket* AND bond* AND review) OR TS = (bracket* AND bond* AND “systematic review”) OR TS = (bracket* AND bond* AND “meta-analysis”) NOT TS = (letter OR editorial OR comment)
Cochrane	bracket* AND bond* AND orthodontic*

**Table 2:** This table summarizes bibliographic characteristics, evidence settings, number of included primary studies, and the outcome definition and measurement framework of transfer accuracy across the included systematic reviews.

First Author	Year	Journal	Review Type	Evidence Setting	No. of Primary Studies (Qualitative)	Outcomes and Measurement Framework
Sabbagh et al. [11]	2022	Journal of Clinical Medicine	Systematic review and meta-analysis	Mixed (in vivo and ex vivo)	16	Bracket transfer accuracy quantified as linear and angular mean transfer errors (MTE), comparing planned vs. achieved bracket positions, meta-analysis performed on ex vivo data.
Bakdach et al. [6]	2022	International Orthodontics	Systematic review and meta-analysis	Mixed (in vitro and in vivo)	7	Linear (mesiodistal, vertical, buccolingual) and angular (angulation, torque, rotation) transfer errors measured via superimposition of original setup vs. final bracket positions.
Campobasso et al. [5]	2023	International Journal of Dentistry	Systematic review and meta-analysis	Mixed (in vitro and in vivo, predominantly in vitro)	15	Linear and angular transfer inaccuracy (MD/OG/BL; torque/tip/rotation) assessed in digital indirect bonding with 3D-printed customized devices, 7 studies included in quantitative synthesis.
Palone et al. [12]	2023	European Journal of Orthodontics	Systematic review and meta-analysis	Mixed (in vitro and in vivo)	13	Accuracy quantified as linear (MD/vertical/BL) and angular (tip/torque/rotation) errors derived from digital superimpositions of planned vs. transferred bracket positions, includes comparative analysis hard vs. soft trays.

**Table 3:** AMSTAR-2.

AMSTAR-2 Domain	Sabbagh et al. [11]	Palone et al. [12]	Campobasso et al. [5]	Bakdach et al. [6]
1. Research question includes PICO components	Yes	Yes	Yes	Yes
2. Protocol registered before review conduct	Yes	Yes	Yes	No
3. Explanation for inclusion of study designs	Yes	Yes	Yes	Yes
4. Comprehensive literature search strategy	Yes	Yes	Yes	Yes
5. Study selection performed in duplicate	Yes	Yes	Yes	Yes
6. Data extraction performed in duplicate	Yes	Yes	Yes	Yes
7. List of excluded studies provided	No	Yes	No	No
8. Description of included studies adequate	Yes	Yes	Yes	Yes
9. Risk of bias assessment appropriate	Yes	Yes	Yes	Yes
10. Funding sources of primary studies reported	No	No	No	No
11. Appropriate meta-analysis methods used	Yes	Yes	Yes	Yes
12. Impact of risk of bias considered in synthesis	Yes	Yes	Partial Yes	Yes
13. Risk of bias considered in interpretation	Yes	Yes	Yes	Yes
14. Heterogeneity adequately investigated	Yes	Yes	Yes	Yes
15. Publication bias assessed	Yes	Yes	No	Yes
16. Conflict of interest declared	Yes	Yes	Yes	Yes

**Table 4:** Summary of primary study overlap across included systematic reviews. Full overlap matrix is provided in Supplementary Table S1.

Systematic Review	Primary Studies Included (*n*)	Unique to This Review (*n*)	Shared with ≥1 Other Review (*n*)
Sabbagh et al. [11]	16	8	8
Campobasso et al. [5]	15	1	14
Palone et al. [12]	13	4	9
Bakdach et al. [6]	7	0	7
Total unique primary studies	26	—	—
CCA	0.35 (very high overlap)	—	—

CCA = Corrected Covered Area. Values > 0.15 indicate very high overlap [22]. Full primary study-level overlap matrix: Supplementary Table S1.

**Table 5:** Methodological architecture and outcome construction of transfer accuracy across included systematic reviews. Each row represents a distinct methodological domain; each column represents one included systematic review. Entries indicate presence (Yes), absence (No), or partial presence (Partial Yes) of each methodological characteristic. Abbreviations: 2D = two-dimensional; 3D = three-dimensional; CAD/CAM = computer-aided design/computer-aided manufacturing; ICC = intraclass correlation coefficient.

Methodological Domain	Sabbagh et al. [11]	Bakdach et al. [6]	Campobasso et al. [5]	Palone et al. [12]
Explicit conceptual definition of transfer accuracy provided	Yes	No	No	Yes
Operational definition based on geometric deviation metrics	Yes	Yes	Yes	Yes
Measurement construct type (linear and angular deviations)	Yes	Yes	Yes	Yes
Composite accuracy index used	No	No	No	No
Predominant dimensional measurement framework	3D	3D	Mixed 2D–3D	3D
Dimensional heterogeneity acknowledged	Yes	Yes	Yes	Yes
Reference system explicitly defined	Yes	No	No	Yes
Reference system impact on measurements evaluated	Yes	No	No	Yes
Measurement technologies and software environments heterogeneous	Yes	Yes	Yes	Yes
Standardized measurement pipeline identified	No	No	No	No
Measurement reliability formally assessed at review level	No	No	No	No
Measurement uncertainty quantified or modeled	No	No	No	No
Clinical acceptability thresholds referenced	Yes	Yes	Yes	Yes
Clinical thresholds empirically validated	No	No	No	No
Outcome linked to clinical outcome measures	No	No	No	No
Quantitative synthesis performed	Yes	Yes	Yes	Yes
Narrative synthesis used to address methodological heterogeneity	Yes	Yes	Yes	Yes
Outcome-specific methodological validity evaluated	No	No	No	No
Outcome interpreted as geometric measurement construct	Yes	Yes	Yes	Yes
Outcome interpreted as clinically validated endpoint	No	No	No	No

**Table 6:** Taxonomy of measurement architecture classes across included systematic reviews and underlying primary studies. Each class represents a structurally distinct measurement paradigm. Assignment is based on the dimensional framework and reference system reported or inferable from the included systematic reviews. Studies not assignable to a single class due to mixed or unreported methodology are classified as “Mixed/Unreported”.

Architecture Class	Reference System	Dimensional Framework	Systematic Reviews
Tooth-based 3D	Individual tooth sperimposition	3D (linear + angular)	Sabbagh et al. [11]; Palone et al. [12]
Arch-based 3D	Full arch superimposition	3D (linear + angular)	Sabbagh et al. [11]; Bakdach et al. [6]; Palone et al. [12]
Tooth-based 2D	Individual tooth superimposition	2D projected (planar axes only)	Campobasso et al. [5]; Bakdach et al. [6]
Arch-based 2D	Full arch superimposition	2D projected (planar axes only)	Campobasso et al. [5]
Mixed/Unreported	Not explicitly stated or mixed across sub-studies	Mixed or not reported	All four reviews [5,6,11,12]

**Table 7:** Hierarchical analysis of methodological heterogeneity in transfer accuracy measurement across included systematic reviews. Each row represents one level of the hierarchical model (Figure 2). Abbreviations: 2D = two-dimensional; 3D = three-dimensional; AI = artificial intelligence; CAD/CAM = computer-aided design/computer-aided manufacturing; ICP = iterative closest point registration.

Level	Domain	Key Components	Impact
Input	Methodological Domain	Heterogenous outcome definitions	Introduces systematic variability at foundational measurement level
		Non-equivalent reference systems (tooth-based vs. arch-based)	
		Variable dimensional frameworks (2D, 3D, mixed)	
		Workflow dependent pipelines	
Mechanism	Structural Mechanism	(software, registration, segmentation)	Creates fundamental incompatibility between studies
		Methodological non-comparability of transfer accuracy measurements	
Epistemic	Epistemic Consequences	Fragmentation of outcome construct	Undermines validity of meta-analyses and systematic reviews
		Structurally non-equivalent evidence	
		Non-cumulative evidence synthesis	
		Inconsistent interpretative thresholds	
Clinical	Clinical Implications	Lack of generalizable interpretation	Restrict evidenced based clinical decision making
		Transfer accuracy as procedural performance metric vs. clinically validated outcome	
		Limited clinical translatability	

## Data Availability

The data presented in this study are available within the article and its Supplementary Materials. The predefined data extraction framework and coding structure used for construct-level methodological mapping are provided as Supplementary Tables S1 and S2. The full primary study overlap matrix across included systematic reviews is provided as Supplementary Table S1. No additional datasets were generated or analyzed beyond those presented in the manuscript and supplementary files.

## Supplementary Materials

The following supporting information can be downloaded at: https://www.mdpi.com/article/10.3390/bioengineering13060607/s1, Table S1: Overlap matrix of primary studies across included systematic reviews. Each cell marked ‘X’ indicates inclusion of the corresponding primary study in the respective systematic review; Table S2: Predefined data extraction framework and coding structure used for construct-level methodological mapping of transfer accuracy across included systematic reviews.
