# Supplementary material for: Transfer Accuracy in Digital Indirect Bonding: A Methodological Umbrella Review of Definitions, Measurement Frameworks, and Evidence Synthesis
Source: Bioengineering (Basel). 2026 May 23;13(6):607. doi: 10.3390/bioengineering13060607 (PMC13295476; doi:10.3390/bioengineering13060607)
Supplement: Supplementary file 1 [file bioengineering-13-00607-s001.zip › bioengineering-4307441-supplementary.pdf]

Supplementary Table S1. Overlap matrix of primary studies across included systematic reviews. Each cell marked 'X' indicates inclusion of the corresponding primary study in the respective systematic review.

Primary Study	Sabbagh et al.	Campobasso et al.	Palone et al.	Bakdach et al.
Aguirre (2006)	X	X	X	
Castilla (2014)	X	X	X	X
Grunheid (2016)	X	X	X	X
Kim (2012)	X	X	X	X
Zhang (2019)	X	X	X	X
Nishimori (2021)	X	X	X	X
Park (2021)	X	X	X	X
Jyothikiran (2014)	X	X	X	X
Hodge (2004)	X	X	X	
Jung (2014)	X	X	X	
Kamanirmala (2017)	X	X	X	
Shaikh (2013)	X	X	X	
Mehta (2021)	X	X	X	
Garg (2018)	X	X	X	
Son (2011)		X		
Ciuffolo (2006)	X			
Foley (2001)	X			
Guenthner (2007)	X			
Hickman (2008)	X			
Koo (2015)	X			
Tavares (2003)	X			
Wendt (2012)	X			
Niazi (2023)			X	
Kolioti (2023)			X	
Bhuwad (2023)			X	
Koch (2022)			X	

Abbreviations: CCA = Corrected Covered Area. CCA = 2S / (N × (R − 1)), where S = total number of study-review inclusions minus the number of unique studies, N = number of unique studies, and R = number of reviews. A CCA of 0–0.05 indicates slight overlap; 0.06–0.10 moderate; 0.11–0.15 high; >0.15 very high [22].

Supplementary Table S2. Predefined data extraction framework and coding structure used for construct-level methodological mapping of transfer accuracy across included systematic reviews. Variables were extracted at both review level and synthesized primary-study methodological level, depending on the analytical domain. The framework was specifically designed to characterize conceptual definitions, measurement constructs, dimensional frameworks, reference systems, technological environments, reliability considerations, interpretative models, and methodological synthesis strategies related to transfer accuracy operationalization. All variables were extracted independently by two reviewers (E.L.; A.V.); discrepancies were resolved through discussion and consensus. Inter-reviewer agreement was assessed using Cohen's kappa (κ = 0.89).

Domain	Variable	Coding Rules / Response Options
**Bibliographic**	First author	Free text
	Year of publication	Numeric (yyyy)
	Journal	Free text
	Review type	Systematic review / Systematic review with meta-analysis
	PROSPERO registration	Yes / No
**Evidence base**	Evidence setting	In vitro / In vivo / Ex vivo / Mixed
	Number of primary studies (qualitative synthesis)	Numeric
	Number of primary studies (quantitative synthesis)	Numeric
	Publication range of primary studies	Numeric range (yyyy–yyyy)
**Conceptual definition**	Explicit conceptual definition of transfer accuracy provided	Yes / No
	Definition text (verbatim or paraphrased)	Free text
	Definition based on geometric deviation metrics	Yes / No
**Measurement construct**	Linear deviation metrics reported	Yes / No
	Angular deviation metrics reported	Yes / No
	Composite accuracy index used	Yes / No; if yes, specify
	Deviation axes reported (linear)	Mesiodistal / Buccolingual / Vertical / Combined / Not specified
	Deviation axes reported (angular)	Torque / Angulation / Rotation / Combined / Not specified
**Dimensional framework**	Predominant dimensional framework	2D / 3D / Mixed 2D–3D / Not specified
	Dimensional heterogeneity among primary studies acknowledged	Yes / No
	2D and 3D data pooled in same synthesis	Yes / No / Not applicable
**Reference system**	Reference system explicitly defined	Yes / No
	Reference system type	Tooth-based / Arch-based / Mixed / Not specified
	Impact of reference system on measurements evaluated	Yes / No
	Multiple reference systems present across primary studies	Yes / No
**Measurement technology**	Imaging/acquisition modality	Intraoral scanner / CBCT / Digitized physical model / Mixed / Not specified
	Software environment reported	Yes / No; if yes, specify
	Registration algorithm reported	Yes / No; if yes, specify (ICP / landmark-based / surface-based / hybrid / not specified)
	Coordinate system definition reported	Yes / No
	Standardized measurement pipeline identified	Yes / No
**Reliability and uncertainty**	Intra-rater reliability reported	Yes / No; if yes, specify metric (ICC / kappa / TEM / other)
	Inter-rater reliability reported	Yes / No; if yes, specify metric
	Measurement uncertainty quantified	Yes / No; if yes, specify method (SD / Bland–Altman / expanded uncertainty)
	Measurement uncertainty modeled or propagated	Yes / No

Domain	Variable	Coding Rules / Response Options
**Clinical interpretability**	Clinical acceptability thresholds referenced	Yes / No
	Threshold values reported	Free text (mm / degrees)
	Threshold origin reported	Yes / No; if yes: ABO / Andrews / other / not specified
	Thresholds empirically validated	Yes / No
	Outcome linked to clinical outcome measures	Yes / No; if yes, specify outcome
**Synthesis strategy**	Quantitative synthesis performed	Yes / No
	Meta-analysis method	Fixed effects / Random effects / Not specified
	Narrative synthesis used	Yes / No
	Methodological heterogeneity addressed in synthesis	Yes / No; if yes, specify method (subgroup / sensitivity / narrative)
**Interpretative framing**	Outcome interpreted as geometric measurement construct	Yes / No
	Transfer accuracy interpreted as clinically validated outcome	Yes / No
	Outcome-specific methodological validity evaluated	Yes / No
**Quality appraisal**	AMSTAR-2 applied	Yes / No
	Overall AMSTAR-2 rating	High / Moderate / Low / Critically low
	Critical domain weaknesses identified	Yes / No; if yes, specify domain

Abbreviations: ABO = American Board of Orthodontics; CBCT = cone beam computed tomography; ICC = intraclass correlation coefficient; ICP = iterative closest point; SD = standard deviation; TEM = technical error of measurement.
